# Secondary cluster headache and numb chin syndrome as initial manifestation of high-grade B-lymphoma: a case report

**DOI:** 10.1186/s13256-021-03016-9

**Published:** 2021-09-14

**Authors:** Joe Munoz-Cerón, Felipe Díaz-Forero, Adriana Buitrago, Sandra Chinchilla

**Affiliations:** 1 Present Address: Departamento de Neurologia, Hospital Univesitario Mayor MEDERI - Universidad del Rosario, Bogotá, Colombia; 2 Departamento de Neurologia , Clinica Univesitaria Colombia - Keralty, Bogotá, Colombia; 3 Deparatmento de Medicinia Nuclear - Hospital Univesitario Mayor MEDERI, Universidad del Rosario, Bogotá, Colombia; 4 Instituto Nacional de Cancerologia - Compensar, Bogotá, Colombia

**Keywords:** Cluster headache, Lymphoma, Etiologies, Secondary headache, Cancer

## Abstract

**Background:**

Cluster headache is a primary condition characterized by severe headache accompanied by trigeminal autonomic signs. By definition, it is not attributed to underlying etiologies; however, under certain clinical characteristics, secondary etiologies must be ruled out.

**Case presentation:**

We present the case of a 48-year-old Hispanic man with a history of episodic right orbital pain, lasting 30 minutes, associated with ipsilateral tearing, who prior to the onset of his symptoms reported loss of appetite, weight loss, and paresthesias in the right chin region. After work-up studies, high-grade lymphoma with infiltration to the right submental nerve was diagnosed, in which numb chin syndrome was the initial presentation. Despite initiation of treatment, the patient died 3 weeks after the diagnosis.

**Conclusions:**

In the study of cluster headache, underlying etiologies must be considered when there are atypical clinical manifestations. Within these etiologies, metastases to pericranial nerves must be included, which, besides generating localized symptoms, can activate the trigeminal vascular system simulating headaches of primary etiology.

## Background

Although by definition cluster headache is a primary entity, its clinical approach includes ruling out secondary etiologies. Based on the classification scheme of the International Headache Society, secondary etiologies of vascular origin, nonvascular intracranial causes by cerebrospinal fluid flow disorders and by neoplastic lesions have been described [1–3].

Although cases related to the compromise of other cranial structures such as the sinuses and the ocular region have been reported [4], there are no descriptions of cases, to the best of our knowledge, in which cranial involvement by pericranial nerve compromise is demonstrated in the context of systemic diseases. We report a case in which high-grade lymphoma with invasion to the submental nerve was diagnosed, with a manifestation of numb chin syndrome and symptomatic cluster headache.

## Case presentation

A Hispanic 48-year-old man referred to the specialized headache consultation for diagnosis of cluster headache, which did not obtain improvement of symptoms after treatment with oxygen at 10 L per minute, verapamil 240 mg, topiramate 100 mg per day, and bilateral occipital block with lidocaine 2% without epinephrine. In our assessment, we found a 4-month history of high-severity, 30-minute right orbital pain episodes associated with ipsilateral tearing without residual pain, as often as two to eight attacks per day; precipitating factors were not identified. There was no history of headache prior to the referred period, and no smoking or alcohol consumption. The psychosocial and family history was irrelevant. In this evaluation, there was also a documented report of paresthesias in the right submental region together with reports of 8-pound weight loss attributed by the patient to a decrease in food intake since the beginning of the headache episodes. The general physical examination showed slight induration in the gingival region corresponding to the territory of the right submental nerve; the neurological exploration was within normal limits.

**Fig. 1:**
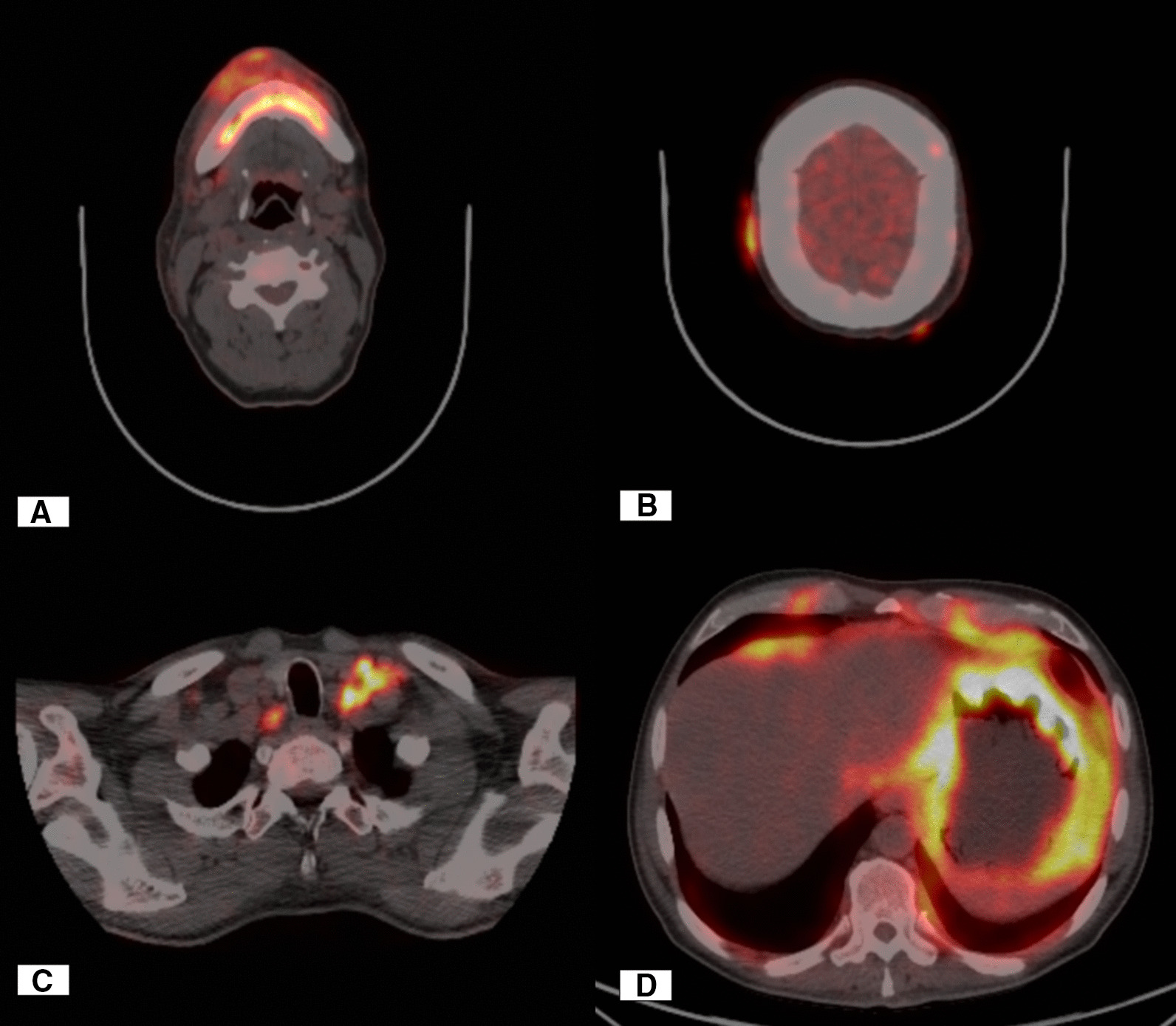
Images (18F-FDG-PET). **A** Hypermetabolic zone in soft tissues of the right lower jaw region, plus the ipsilateral submental nerve. **B** Hypermetabolic nodular lesions of subcutaneous location in the bilateral parietal region. **C** Hypermetabolic lesions suggestive of nodular cluster of the left internal jugular chain and right paratracheal adenopathy. **D** Axial image of abdomen with distended stomach. Thickening of the gastric wall is observed, which presents major metabolism throughout it

**Fig. 2:**
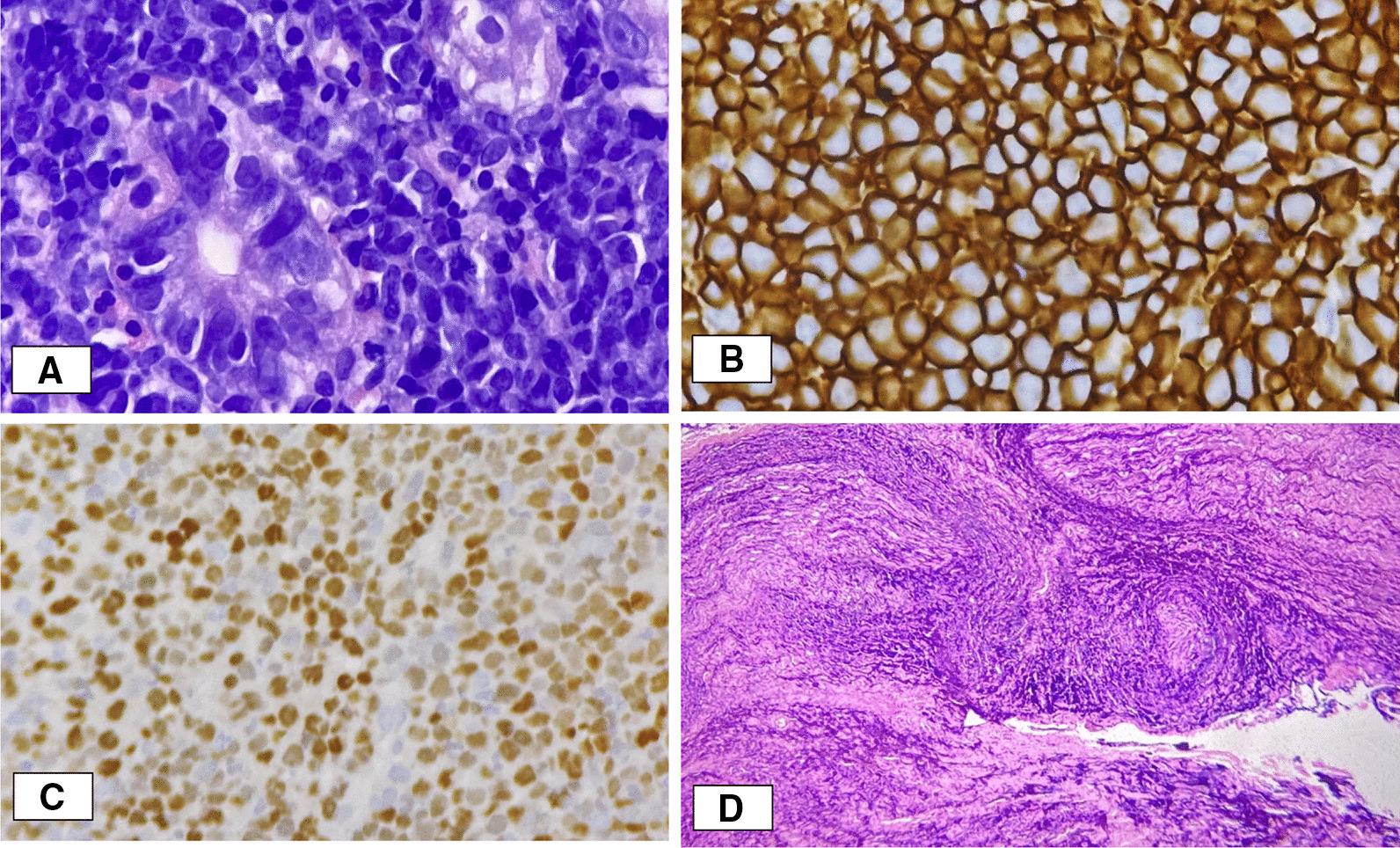
Histopathological findings. **A **[40×, hematoxylin and eosin (HE)]. Gastric mucosa with lymphoid neoplasm consisting of intermediate to large cells with scant cytoplasm arranged in a diffuse pattern. **B **(40×, immunohistochemistry). The neoplastic cells show immunoreactivity for CD20. **C **(40×, immunohistochemistry). The proliferation rate is very high, with nearly 100% of the cells positive for Ki67.** D** (10×, HE). Soft tissues and nerve fillets of maxillary region with involvement by lymphoid neoplasm

Due to the refractoriness of the symptoms along with previous Magnetic resonance imaging (MRI), Magnetic resonance angiography (MRA), Magnetic resonance venography (MRV), and Cerebro spinal fluid (CSF) studies within normal limits in addition to weight loss, it was considered to rule out underlying systemic etiologies with neurological manifestations. F-2-fluoro-2-deoxy-d-glucose positron emission tomography (18F-FDG-PET) was then performed, which demonstrated findings suggestive of disseminated metastatic disease with predominance in the gastric region, affecting right mandibular region plus multiple foci in the cranial region (Fig 1). Due to the predominant gastric involvement, the patient was referred to gastroenterology where an endoscopy of digestive tract was performed, of which leather bottle stomach was reported with histology study of high-grade B-cell lymphoma. This finding matched the result of biopsy performed in the right submental nerve over the induration area reported at our service admission (Fig 2). After the excisional biopsy on the mental nerve, the pain episodes disappeared, leaving a hypoesthetic area in the corresponding territory. After the above-described clinical analysis, the diagnosis of cluster headache and numb chin syndrome secondary to B-cell lymphoma stage 4B was considered. Despite initiation of treatment, the patient died 3 weeks after the diagnosis.

## Discussion and conclusion

According to the International Classification of Headache Disorders (ICHD-3), cluster headache is characterized by the presence of at least five episodes of high-severity pain, most of the time in the orbital region with at least one trigeminal autonomic symptom (Table 1). This entity is more frequent in men, and its onset is usually described between 20 and 40 years of age [5]. The likelihood of secondary etiologies increases when the clinical characteristics do not meet the ICHD 3 diagnostic criteria regarding number, duration of episodes, accompanying symptoms, and frequency of attacks or when, despite meeting them, the patient presents an atypical clinical course in addition to an unsatisfactory therapeutic response. When we face this clinical scenario, it is appropriate to take into account criterion E of the International Classification of Headache, which recommends looking for another entity included in the ICHD-3 that can better explain the signs and symptoms of the patient under study. Although the use of the ICHD-3 criteria has been shown to be useful in the differentiation of primary versus symptomatic etiologies [6], its application in patients with cluster headache of secondary origin has reported accuracy limitations because a significant percentage of these cases may meet the criteria satisfactorily despite demonstrating an underlying etiology [7].

**Table 1.** ICHD 3 diagnostic criteria for cluster headache [5]

Criteria	Description
A.	At least five attacks fulfilling criteria B–D
B.	Severe or very severe unilateral orbital, supraorbital, and/or temporal pain lasting 15–180 minutes (when untreated)
C.	Either or both of the following: (1) at least one of the following symptoms or signs, ipsilateral to the headache: conjunctival injection and/or lacrimation nasal congestion and/or rhinorrhea eyelid edema forehead and facial sweating miosis and/or ptosis (2) a sense of restlessness or agitation
D.	Occurring with a frequency between one every other day and eight per day
E.	Not better accounted for by another ICHD-3 diagnosis

In the reported patient, the criteria A–D were satisfactorily met. Additionally, the MRI, MRV, MRA, CT, and CSF studies were found within normal limits. This analysis initially led to consideration of primary etiology; however, due to the absence of therapeutic response and the presence of weight loss and induration sensation in the territory of the submental nerve, systemic etiologies were ruled out.

High-grade B-cell lymphoma represents a subtype of non-Hodgkin lymphoma that affects the central nervous system (CNS) in 5% of relapses, with a manifestation in the early course of the disease and representing a poor clinical prognostic factor [8].

Numb chin syndrome is characterized by the appearance of sensory symptoms in the territory of the mental nerve, accompanied in some cases by motor symptoms. Although benign etiologies have been described for this syndrome, most of the cases represent the initial symptom of neoplastic diseases [9]. In this case, high-grade B-cell lymphoma was shown, with metastatic lesions in periosteum, jaw region, and right submental nerve, which, due to their size, were not detected in the routine brain images, requiring 18F-FDG-PET, which, due to its ability to detect metabolic changes, has better diagnostic performance compared with conventional neuroimaging. We raised the hypothesis that lesions on the submental nerve can explain the activation of the trigeminovascular system allowing the onset of symptoms that simulate the clinical characteristics suggestive of cluster headache. This model is taken from reports in which head injury can modify neurotransmitters triggering cluster-like symptoms [10]. This hypothesis could be also supported in the control of pain episodes after the nerve resection during the biopsy.

This report highlights the importance of considering neoplastic etiologies with metastases to the cranial nerves as a cause of symptomatic cluster headache. It additionally mentions the need to consider underlying etiologies in the study of cluster headache even when the ICHD-3 criteria regarding number, duration of episodes, and accompanying symptoms are satisfactorily met. Taking into account that the cause of the headache was shown to be related to cranial bone metastasis, this patient can be categorized as secondary headache 11.9 according to the International Classification of Headache.

## Clinical implications

- Although the ICHD 3 criteria in number of episodes, duration of episodes, and accompanying symptoms are met, it is important to consider underlying etiologies, particularly when the clinical course is not as expected.
- It is important to consider neoplastic etiologies with metastases to the cranial nerves as a cause of symptomatic cluster headache.

## Data Availability

Not applicable in this section.

## Abbreviations

ICHD: International Classification of Headache Disorders
MRI: Magnetic resonance imaging
LP: Lumbar punction
CSF: Cerebrospinal fluid
MRA: Magnetic resonance angiography
MRV: Magnetic resonance venography
FDG-PET: Fluorodeoxyglucose–positron emission tomography
